# Impact of Laser Lipolysis With and Without Liposuction on Arm Circumference: A Systematic Review and Meta-Analysis

**DOI:** 10.1093/asjof/ojaf097

**Published:** 2025-09-08

**Authors:** Zhen Yu Wong, Veylamuthen Murugan, Zhen Ning Wong, Pojsakorn Danpanichkul, Ryan Faderani, Muholan Kanapathy, Afshin Mosahebi

## Abstract

Laser-assisted lipolysis (LAL) for arm fat reduction has gained popularity compared with traditional liposuction. The authors of this study aim to quantify changes in arm circumference through LAL and compare outcomes between treatments with and without suction. A Preferred Reporting Items for Systematic Reviews and Meta-Analyses–compliant systematic review was conducted from inception until May 2024, and meta-analysis was performed using Stata. Mean differences in arm circumference were pooled using the DerSimonian and Laird random-effects model. Out of 135 screened studies, 7 were included in the analysis. The pooled arm circumference reduction (*n* = 199) was 2.95 cm (*P* < .001, 95% CI, 1.50-4.41). Subgroup analysis revealed that the reduction with suction was 3.39 cm (*P* = .078, 95% CI, −0.38 to 7.16), and without suction, it was 2.04 cm (*P* = .022, 95% CI, 0.30-3.78). Overall, both clinicians and patients reported high satisfaction levels with the treatment, although satisfaction was notably lower among patients with more advanced conditions. Reported complications were mild and transient, including instances of ecchymosis and prolonged edema. Although the current evidence is limited by small sample size, the safety profile of LAL is favorable and the outcomes are promising. Further studies are needed to validate these findings.

**Level of Evidence: 3 (Therapeutic)**  
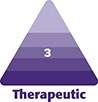

In recent years, numerous fat-reduction procedures have emerged to meet the growing demand for less invasive approaches that offer reduced risk while achieving favorable results.^1^ These procedures include external ultrasound, low-level external laser, injection lipolysis, cryolipolysis, external radiofrequency energy, and percutaneous laser lipolysis.^2-4^ Laser lipolysis has emerged as an alternative surgical approach to traditional liposuction, offering the potential for skin tightening and fat reduction through laser energy, and has been performed both with and without concurrent suction lipectomy. In this technique, laser energy is delivered through an optical fiber targeting the subcutaneous fat layer. This leads to adipocyte membrane damage, resulting in the leakage of cellular contents into the interstitial compartment and potentially irreversible adipocyte collapse.^5^ The interstitial fatty debris can either be cleared by applied suction or processed by the body's innate metabolic mechanisms. It remains comparatively invasive because of the need for subcutaneous instrumentation.

Contouring of the upper arms remains a challenging area in body sculpting. Traditional liposuction is effective for volume reduction but may exacerbate skin laxity in patients with poor dermal elasticity, leading to unsatisfactory cosmetic outcomes. On the contrary, brachioplasty, although effective in managing severe laxity, is associated with conspicuous scarring and higher complication rates. Laser-assisted lipolysis (LAL) offers a potential solution to this dilemma by delivering laser energy through an optical fiber into the subcutaneous fat layer, inducing both fat disruption and tissue contraction. This synergistic effect may make LAL particularly suitable for arm contouring, especially in patients with mild-to-moderate skin laxity where conventional methods are suboptimal.

Despite the increasing popularity of LAL, there is limited evidence quantifying its effectiveness in reducing arm circumference. Understanding the extent of arm circumference reduction following LAL is crucial for evaluating its clinical utility. Additionally, the impact of performing LAL with or without suction assistance remains unclear. This systematic review aims to critically analyze existing research on LAL, focusing on quantifying arm circumference reduction as the primary outcome. A secondary objective is to compare outcomes between suction-assisted and nonsuction LAL techniques in terms of safety, efficacy, and patient satisfaction. By synthesizing the available evidence, this review seeks to contribute to the ongoing discourse on the optimal application of LAL and inform best practices in body contouring procedures.

## METHODS

### Search Strategy and Selection Criteria

This review was conducted in accordance with the Cochrane Handbook for Systematic Reviews of Interventions and adheres to the Preferred Reporting Items for Systematic Reviews and Meta-Analyses (PRISMA) guidelines.^6^

A prespecified protocol was prospectively registered on the PROSPERO database (CRD42024554351). Because the review did not involve the use of identifiable patient data, institutional ethical approval was not required. A systematic search of the literature was performed across MEDLINE and Embase from inception to May 23, 2024, without language restrictions. Keywords and subject headings used in the search included combinations of terms such as “laser lipolysis,” “laser-assisted liposuction,” “arm circumference,” and “fat reduction” (Supplemental Table 1).

Inclusion criteria included patients aged over 18 years old, the intervention being LAL measuring arm circumference, and outcomes of circumference reduction. Studies were excluded if they did not report extractable data on changes in arm circumference, were systematic reviews, lacked full-text access, or focused on pediatric populations. Two reviewers independently screened titles and abstracts, followed by full-text assessments for eligibility. Arm circumference was selected as the primary outcome because of its consistent use across studies as a measure of treatment effectiveness. Patient satisfaction and adverse events were extracted as secondary outcomes, given their clinical importance and frequent documentation in the included literature.

### Data Extraction

Data were extracted using a predesigned, standardized template. Key information collected included study design, country, sample size, participant demographics (age and sex), LAL technique (with or without suction), duration of follow-up, the primary outcome being mean change in arm circumference, and relevant classification systems, including Teimourian grading where reported. Two reviewers independently verified all extracted data. Secondary outcomes, including complications and patient satisfaction, were summarized narratively because of variability in reporting formats. The Teimourian grading system classifies upper arm contour deformities based on the degree of fat accumulation and skin laxity to guide treatment selection, ranging from Grade I (minimal fat, no laxity) to Grade IV (severe excess with significant laxity).

### Risk of Bias Assessment

The methodological quality of the included cohort studies and case series was assessed using the Joanna Briggs Institute Critical Appraisal Tools.^7^ Each study was evaluated across defined domains and subsequently categorized as having low, moderate, or high risk of bias (reported as good, fair, or poor quality).

### Statistical Analysis

Meta-analysis was performed using Stata version 14 (StataCorp, College Station, TX) and Review Manager version 5.3 (Cochrane Collaboration, London, United Kingdom). All pooled analyses used the DerSimonian and Laird random-effects model, selected irrespective of heterogeneity, in line with recommendations favoring its robustness over fixed-effects models.^8-10^ For continuous outcomes, mean differences and corresponding 95% CIs were calculated. When studies lacked reported means or standard deviations, estimates were derived using the method described by Cai et al.^11^ Data reported in different units were converted using established conversion factors for comparability. In instances where raw data were not available, study authors were contacted. If no response was received, data were extracted from graphical figures using Engauge Digitizer software (version 12.0).

## RESULTS

**Figure 1.**
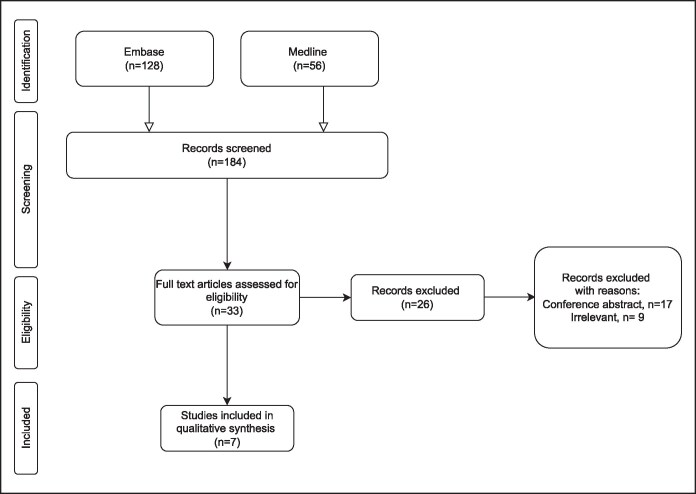
Preferred Reporting Items for Systematic Reviews and Meta-Analyses flow diagram illustrating the study selection process for the systematic review and meta-analysis.

The initial database search yielded 184 articles. After removing 49 duplicates, 135 articles remained for title and abstract screening. Of these, 33 articles were assessed in full-text screening, and 7 studies met the inclusion criteria and were included in the final analysis (Figure 1), encompassing 199 patients.^12-18^ The included studies displayed notable variation in laser types, energy levels, and study designs. Three case series, 3 prospective cohort studies and 1 randomized controlled trial were included in this study (Table 1). Suction-assisted LAL was performed using standard liposuction cannulas in some studies, whereas others used manual expression or no suction at all. Follow-up periods ranged from 2 weeks to 6 months across studies. BMI was inconsistently reported but ranged from 24.6 to 32.96, corresponding to normal-to-overweight classifications. The mean participant age across studies ranged from ∼35 to 54.6 years. The risk of bias assessment is provided in Supplemental Table 2. The overall risk of bias was predominantly moderate to high.

**Table 1.** Summary of Key Characteristics of the Studies Included in the Systematic Review, Including Study Design, Sample Size, Intervention Type, and Outcome Measures

Study	Type of laser	Power level	Energy level	Device(s)	Suction and no suction (S/NS/both)	Follow-up time	Country	Study design	Total sample	Gender	BMI (kg)	Age (mean, SD, years)
Dudelzak (2009)^12^	1064 nm Nd:YAG laser	10 W	7112-12,026 J	SmartLipo	Both	6 months	USA	Prospective cohort study	20	Female	NR*	54.2
Kotlus and Mok (2011)^12^	1320 nm Nd:YAG pulsed laser	15 W	NR*	CoolLipo	NS	3 months	USA	Case series	5	Female	78.2	35 (28-45)
Leclère et al (2015)^17^	1470 nm diode laser	15 W	15-18 kJ	Alma Lasers	S	6 months	France	Prospective cohort study	90	NR*	25.8 ± 1.2	48.9 ± 10.4
Leclère et al (2016)^18^	1470 nm diode laser	15 W	15-18 kJ	Alma Lasers	S	6 months	France	Prospective cohort study	44	NR*	25.8 ± 1.4	45.3 ± 10.2
Nestor et al (2012)^14^	5 × 635 nm diode laser	17 mW	3.94J/cm^2^	Zerona	NS	2 weeks	USA	Randomized controlled trial	20	NR*	27.96 ± 5	43.75 ± 10.37
Nilforoushzadeh et al (2023)^15^	Endolift laser	5-8 W	NR*	LASEMAR1500	NS	3 months	Iran	Case series	10	Male & female	NR*	25-55
Nicoli et al (2015)^16^	1470 nm diode laser	15W	150 mJ	LASEMAR1500	NS	6 months	Taiwan	Case series	10	Female	NR*	54.6 ± 9.3

*NR, not reported; NS, nonsignificant.

**Figure 2.**
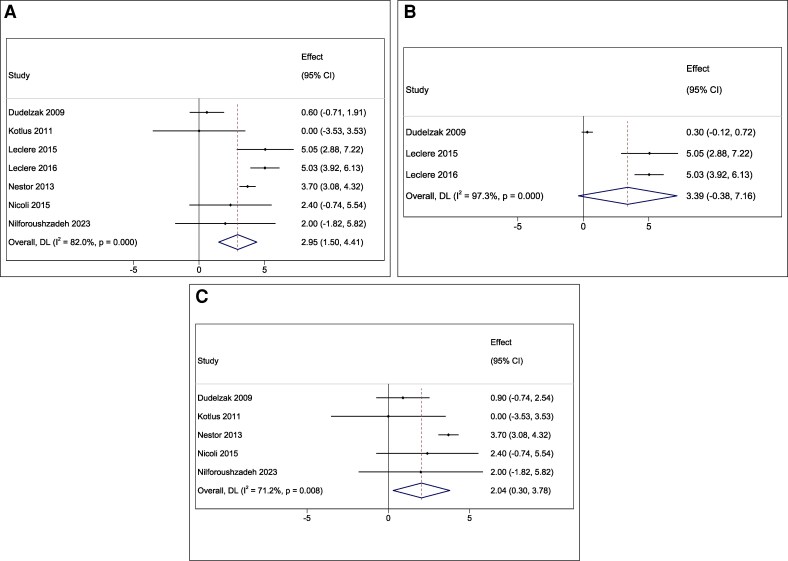
Forest plots showing (A) the overall pooled arm circumference reduction following laser-assisted lipolysis, (B) subgroup analysis of arm circumference reduction with suction-assisted laser lipolysis, and (C) subgroup analysis of arm circumference reduction without suction.

The pooled mean reduction in arm circumference across all studies was 2.95 cm (95% CI, 1.50-4.41, *P* < .001, *I*^2^ = 82.0%; Figure 2A).^12-18^ Subgroup analysis revealed that studies incorporating suction-assisted LAL achieved a pooled mean reduction of 3.39 cm (95% CI, −0.38 to 7.16, *P* = .078, *I*^2^ = 97.3%), which was not statistically significant and demonstrated a significant and substantial degree of heterogeneity (Figure 2B).^12,17,18^ In contrast, studies without suction assistance reported a statistically significant pooled mean reduction of 2.04 cm (95% CI, 0.30-3.78, *P* = .022, *I*^2^ = 71.2%), although there was a significant and substantial degree of heterogeneity (Figure 2C).^12-15^

Notably, only 1 study directly compared suction-assisted and nonsuction techniques and found no significant difference between the 2 approaches.^12^ Grade-specific outcomes revealed that patients with Teimourian Grades I and II experienced more significant reductions in arm circumference and higher satisfaction rates compared with those with Grades III and IV.^17,18^ Furthermore, patients with a lower BMI demonstrated greater reductions in arm circumference.^14^ Overall patient satisfaction was reported to be high across most studies, although assessments were typically based on unstandardized subjective scales and conducted in single-arm designs. Only 1 comparative study by Nestor et al found significantly higher satisfaction in the LAL group compared with the control group. Additionally, beyond fat reduction, LAL was reported to improve skin tightening and tonicity. In a study by Dudelzak et al, subjective assessment revealed clinical improvement in appearance, including circumference reduction and skin retraction or tightening, in 16 treated patients. In one study, the authors confirmed the skin tightening effect using objective skin caliper measurements, reporting a significant reduction in average skin pinch across both arms in patients with Teimourian Grade I (0.7 cm, *P* < .01) and Grade II (2.1-2.9 cm, *P* < .01). In contrast, in another study using subjective assessment, the authors found no improvement in skin tightening or firmness among patients with Teimourian Grade III (*P* = .61) and Grade IV (*P* = .25).

Adverse effects were mild and transient, including edema, ecchymosis, and discomfort, with occasional reports of prolonged edema; no serious or long-term complications were reported. Pain scores during anesthesia and postprocedure discomfort were consistently low across studies, with minimal differences between suction and nonsuction groups. Mean downtime post procedure was minimal, ranging from 0.08 to 0.73 days.

## DISCUSSION

This systematic review is the first comprehensive analysis to quantify the reduction in arm circumference following LAL, providing a consolidated assessment of its efficacy and safety. The pooled data from 7 studies demonstrated that LAL can achieve a significant reduction in arm circumference. Although statistically significant, it remains unclear what degree of reduction in arm circumference is directly correlated with meaningful changes in aesthetic appearance. Moreover, the superiority of 1 method over the other was not established. Furthermore, the consistently high patient satisfaction, notable skin tightening effects, and minimal complications reported across studies are encouraging and support the use of LAL as a viable treatment option.

The notable reduction in arm circumference positions LAL as a promising, minimally invasive option for arm contouring. Beyond fat reduction, LAL offers the added benefit of skin tightening because of laser-induced collagen remodeling.^14^ This dual effect is particularly advantageous for patients seeking body contouring without the extensive recovery associated with traditional liposuction. The combination of fat reduction and skin tightening makes LAL an appealing alternative, especially for individuals with mild-to-moderate adipose tissue accumulation in the arms.^14^ Reported downtime ranged from under 2 to 18 h, reflecting a return to normal activities rather than full recovery. Although this suggests a potential advantage of minimal downtime with LAL, these findings should be interpreted cautiously because of small sample sizes and nonstandardized measurement methods. Further studies with uniform definitions of recovery and objective reporting are needed to validate these outcomes.

The comparison between suction-assisted and nonsuction LAL highlights the importance of treatment selection based on patient-specific factors. The suction technique does not demonstrate superiority; however, this conclusion is limited by the small sample size. This suggests that suction may enhance fat removal efficiency but may not be universally necessary.^19^ Besides, the severity of adiposity and skin laxity, along with patient BMI, likely influence treatment outcomes. Patients with higher BMI or more severe skin laxity may benefit more from suction-assisted LAL, whereas those with lower BMI or milder deformities might achieve satisfactory results with nonsuction techniques.^20^ Future studies should stratify patients by severity and BMI to better tailor treatment approaches. Moreover, the potential accumulation of cellular debris from lysed adipocytes in nonsuction LAL raises concerns about local inflammatory responses. Theoretically, this could increase the risk of seroma formation, fibrosis, or contour irregularities because of delayed clearance and tissue remodeling. However, current clinical evidence remains limited, and no included studies systematically evaluated these long-term complications. Further comparative studies with long-term follow-up are warranted to clarify these risks.

This study's primary strength lies in being the first to systematically quantify arm circumference reduction following LAL, offering valuable insights into its efficacy. Additionally, the inclusion of both suction-assisted and nonsuction techniques provides a comprehensive comparison. However, the small pooled sample size (*n* = 199) and substantial heterogeneity (*I*^2^ = 71%-97%) with a short follow-up period limit the robustness of pooled estimates, warranting cautious interpretation. Most included studies exhibited a moderate-to-high risk of bias, particularly because of methodological limitations such as unclear randomization procedures, lack of blinding, and incomplete outcome reporting, which may affect the reliability of the findings. Furthermore, the lack of standardized outcome measures and incomplete reporting of BMI and gender data may have influenced the pooled outcomes. Future research should prioritize well-designed, low-bias randomized controlled trials with long-term follow-up to assess sustained outcomes and safety.

## CONCLUSIONS

LAL is a safe and effective method for reducing arm circumference, with the additional benefits of skin tightening. Although suction-assisted LAL may offer enhanced fat reduction for certain patients, both suction and nonsuction techniques present promising results. Future high-quality studies are warranted to further investigate the impact of patient BMI and adiposity severity on treatment outcomes and to establish standardized protocols for optimizing LAL in arm contouring.

## Supplementary Material

ojaf097_Supplementary_Data
