# Supplementary material for: Impact of Laser Lipolysis With and Without Liposuction on Arm Circumference: A Systematic Review and Meta-Analysis
Source: Aesthet Surg J Open Forum. 2025 Sep 8;7:ojaf097. doi: 10.1093/asjof/ojaf097 (PMC12415858; doi:10.1093/asjof/ojaf097)
Supplement: ojaf097_Supplementary_Data [file ojaf097_supplementary_data.docx]

**Supplemental Table 1.** Search strategies

Ovid MEDLINE(R) ALL <1946 to May 23, 2024>

| **Step** | **Search Strategy** | **Results** |
| --- | --- | --- |
| 1 | (Lasers or Laser or Q-Switched Lasers or Laser, Q-Switched or Lasers, Q-Switched or Q Switched Lasers or Q-Switched Laser or Pulsed Lasers or Laser, Pulsed or Lasers, Pulsed or Pulsed Laser or Continuous Wave Lasers or Continuous Wave Laser or Laser, Continuous Wave or Lasers, Continuous Wave or Masers or Maser or (laser or laser adapter or laser adaptor or laser associate or laser beam or laser beam delivery unit holder or laser device or laser holder or laser irradiation or laser radiation or laser ray or lasers or radiation, laser or low level laser therapy or endoscopic laser therapy or laser biostimulation or laser therapy or laser therapy, low-level or laser treatment or low energy laser therapy or low energy laser treatment or low intensity laser therapy or low intensity laser treatment or low level laser therapy or low level laser treatment or low level light therapy or low power laser therapy or low power laser treatment or low-level light therapy)).ti,ab. | 332,015 |
| 2 | (Adipos* or Lipo* or fat reduction or contour* or localized fat or subcutaneous fat or fat).ti,ab. | 943,208 |
| 3 | (Arm or upper limb or brachia* or Upper Extremity).mp. [mp=title, book title, abstract, original title, name of substance word, subject heading word, floating sub-heading word, keyword heading word, organism supplementary concept word, protocol supplementary concept word, rare disease supplementary concept word, unique identifier, synonyms, population supplementary concept word, anatomy supplementary concept word] | 301,867 |
| 4 | 1 and 2 | 7,120 |
| 5 | 3 and 4 | 56 |

**Embase <1974 to 2024 May 23>**

| **Step** | **Search Strategy** | **Results** |
| --- | --- | --- |
| 1 | (Lasers or Laser or Q-Switched Lasers or Laser, Q-Switched or Lasers, Q-Switched or Q Switched Lasers or Q-Switched Laser or Pulsed Lasers or Laser, Pulsed or Lasers, Pulsed or Pulsed Laser or Continuous Wave Lasers or Continuous Wave Laser or Laser, Continuous Wave or Lasers, Continuous Wave or Masers or Maser or laser adapter or laser adaptor or laser associate or laser beam or laser beam delivery unit holder or laser device or laser holder or laser irradiation or laser radiation or laser ray or lasers or radiation, laser or low level laser therapy or endoscopic laser therapy or laser biostimulation or laser therapy or laser therapy, low-level or laser treatment or low energy laser therapy or low energy laser treatment or low intensity laser therapy or low intensity laser treatment or low level laser therapy or low level laser treatment or low level light therapy or low power laser therapy or low power laser treatment or low-level light therapy).ti,ab. | 345,685 |
| 2 | (Adipos* or Lipo* or fat reduction or contour* or localized fat or subcutaneous fat or fat).ti,ab. | 1,198,799 |
| 3 | (Arm or upper limb or brachia* or Upper Extremity).mp. [mp=title, abstract, heading word, drug trade name, original title, device manufacturer, drug manufacturer, device trade name, keyword heading word, floating subheading word, candidate term word] | 493,165 |
| 4 | 1 and 2 | 9,297 |
| 5 | 3 and 4 | 128 |

**Supplemental Table 2.** Risk of bias assessment for included case series studies

| CStudy | Level of evidence | Q1 | Q2 | Q3 | Q4 | Q5 | Q6 | Q7 | Q8 | Q9 | Q10 | Total score | Rating |  |
| --- | --- | --- | --- | --- | --- | --- | --- | --- | --- | --- | --- | --- | --- | --- |
| Kotlus 2011 | 4 | Y | Y | N | N | Y | N | N | Y | N | N | 4 | H |  |
| Nilforoushzadeh 2023 | 4 | Y | Y | Y | N | Y | N | N | Y | N | Y | 6 | M |  |
| Nicoli 2015 | 4 | Y | Y | Y | Y | Y | N | N | Y | Y | Y | 8 | L |  |
| Q1) Were there clear criteria for inclusion in the case series?  Q2) Was the condition measured in a standard, reliable way for all participants included in the case series?  Q3) Were valid methods used for identification of the condition for all participants included in the case series?  Q4) Did the case series have consecutive inclusion of participants?  Q5) Did the case series have complete inclusion of participants?  Q6) Was there clear reporting of the demographics of the participants in the study?  Q7) Was there clear reporting of clinical information of the participants in the study?  Q8) Were the outcomes or follow up results of cases clearly reported?  Q9) Was there clear reporting of the presenting site(s)/clinic(s) demographic information  Q10) Was statistical analysis appropriate?  Y – Yes, N – No, U – Unsure  L – Low risk of bias, M – Medium risk of bias, - H – High risk of bias | | | | | | | | | | | | | |  |

Supplementary Table 2b Risk of bias assessment for included cohort studies

| Study | Level of evidence | Q1 | Q2 | Q3 | Q4 | Q5 | Q6 | Q7 | Q8 | Q9 | Q10 | Q11 | Total score | Rating |  |
| --- | --- | --- | --- | --- | --- | --- | --- | --- | --- | --- | --- | --- | --- | --- | --- |
| Dudelzak 2009 | 2b | N | N | Y | N | N | Y | Y | Y | U | U | Y | 5 | H |  |
| Leclere 2015 | 2b | N | N | Y | N | N | Y | Y | Y | U | U | Y | 5 | H |  |
| Leclere 2016 | 2b | N | N | Y | N | N | Y | Y | Y | U | U | Y | 5 | H |  |
| Q1) Were the two groups similar and recruited from the same population  Q2) Were the exposures measured similarly to assign people to both exposed and unexposed groups?  Q3) Was the exposure measured in a valid and reliable way?  Q4) Were confounding factors identified?  Q5) Were strategies to deal with confounding factors stated?  Q6) Were the groups/participants free of the outcome at the start of the study (or at the moment of exposure)?  Q7) Were the outcomes measured in a valid and reliable way?  Q8) Was the follow up time reported and sufficient to be long enough for outcomes to occur?  Q9) Was follow up complete, and if not were the reasons to loss to follow up described and explored?  Q10) Were strategies to address incomplete follow up utilised?  Q11) Was appropriate statistical analysis used?  Y – Yes, N – No, U – Unsure  L – Low risk of bias, M – Medium risk of bias, - H – High risk of bias | | | | | | | | | | | | | | |  |

Supplementary Table 2c Risk of bias assessment for included randomised controlled trials

| **Study** | **Level of evidence** | **Randomization process** | **Deviations from intended interventions** | **Missing outcome data** | **Measurement of the outcome** | **Selection of the reported result** | **Overall Bias** |
| --- | --- | --- | --- | --- | --- | --- | --- |
| Nestor 2013 | 1b | M | L | L | M | M | M |
| L – Low risk of bias, M – Medium risk of bias, - H – High risk of bias | | | | | | | |
